# Extracellular acyl-CoA-binding protein as an independent biomarker of COVID-19 disease severity

**DOI:** 10.3389/fimmu.2024.1505752

**Published:** 2025-01-06

**Authors:** Stephane Isnard, Tsoarello Mabanga, Léna Royston, Carolina A. Berini, Simeng Bu, Orthy Aiyana, Hansen Feng, Bertrand Lebouché, Cecilia T. Costiniuk, Joseph Cox, Guido Kroemer, Madeleine Durand, Jean-Pierre Routy, the Biobanque Québécoise de la COVID-19 (BQC-19)

**Affiliations:** ^1^ Inflammation and Immunity in Global Health Program, Research Institute of the McGill University Health Centre, Montreal, QC, Canada; ^2^ Chronic Viral Illness Service, McGill University Health Centre, Montreal, QC, Canada; ^3^ Division of Infectious Diseases, Geneva University Hospitals, Geneva, Switzerland; ^4^ Department of Family Medicine, Faculty of Medicine and Health Sciences, McGill University, Montreal, QC, Canada; ^5^ Centre for Outcomes Research & Evaluation, Research Institute of the McGill University Health Centre, Montreal, QC, Canada; ^6^ Centre de Recherche des Cordeliers, Equipe labellisée par la Ligue contre le cancer, Université de Paris, Sorbonne Université, Inserm U1138, Institut Universitaire de France, Paris, France; ^7^ Metabolomics and Cell Biology Platforms, Institut Gustave Roussy, Villejuif, France; ^8^ Institut du Cancer Paris CARPEM, Department of Biology, Hôpital Européen Georges Pompidou, assistance publique des hôpitaux de Paris (AP-HP), Paris, France; ^9^ Département de Microbiologie, Infectiologie et Immunologie, Centre de Recherche du Centre Hospitalier de l’Université de Montréal, Montréal, QC, Canada; ^10^ Division of Hematology, McGill University Health Centre, Montreal, QC, Canada

**Keywords:** Acyl-CoA-binding protein, autophagy, COVID-19, SARS-CoV-2, proteomics, BQC-19 biobank

## Abstract

**Background:**

Factors leading to severe COVID-19 remain partially known. New biomarkers predicting COVID-19 severity that are also causally involved in disease pathogenesis could improve patient management and contribute to the development of innovative therapies. Autophagy, a cytosolic structure degradation pathway is involved in the maintenance of cellular homeostasis, degradation of intracellular pathogens and generation of energy for immune responses. Acyl-CoA binding protein (ACBP) is a key regulator of autophagy in the context of diabetes, obesity and anorexia. The objective of our work was to assess whether circulating ACBP levels are associated with COVID-19 severity, using proteomics data from the plasma of 903 COVID-19 patients.

**Methods:**

Somalogic proteomic analysis was used to detect 5000 proteins in plasma samples collected between March 2020 and August 2021 from hospitalized participants in the province of Quebec, Canada. Plasma samples from 903 COVID-19 patients collected during their admission during acute phase of COVID-19 and 295 hospitalized controls were assessed leading to 1198 interpretable proteomic profiles. Levels of anti-SARS-CoV-2 IgG were measured by ELISA and a cell-binding assay.

**Results:**

The median age of the participants was 59 years, 46% were female, 65% had comorbidities. Plasma ACBP levels correlated with COVID-19 severity, in association with inflammation and anti-SARS-CoV-2 antibody levels, independently of sex or the presence of comorbidities. Samples collected during the second COVID-19 wave in Quebec had higher levels of plasma ACBP than during the first wave. Plasma ACBP levels were negatively correlated with biomarkers of T and NK cell responses interferon-γ, tumor necrosis factor-α and interleukin-21, independently of age, sex, and severity.

**Conclusions:**

Circulating ACBP levels can be considered a biomarker of COVID-19 severity linked to inflammation. The contribution of extracellular ACBP to immunometabolic responses during viral infection should be further studied.

## Introduction

Severe acute respiratory syndrome coronavirus 2 (SARS-CoV-2) has caused coronavirus disease 2019 (COVID-19) in over 700 million people since 2019 (1). Clinical manifestations of COVID-19 range from a mild and often asymptomatic presentation to severe and sometimes fatal outcomes in around 1% of cases (2). Factors predicting COVID-19 severity and mortality include advanced age and male sex, low socio-economic status, co-morbidities, immunosuppression, vaccination status, as well as biochemical and radiologic findings (1). Comorbidities such as obesity, diabetes, chronic obstructive pulmonary disease (COPD) and cardiovascular disease (CVD) have a major influence on the risk of developing severe COVID-19, which can occur rapidly and unexpectedly, posing an unresolved challenge for clinicians (3).

Biomarkers fall into two categories, pre-infection (*anticipatory)* and post-infection (*reactive)*, for acquisition and outcomes of COVID-19, respectively (1, 4). The identification of reactive predictors of COVID-19 severity that are also causally involved in disease pathogenesis might improve patient management and contribute to the development of innovative therapy. Reactive markers such as C-reactive protein (CRP), ferritin, and interleukin-6 (IL-6) have been associated with COVID-19 severity as well as other comorbidities (5–7).

Autophagy is a catabolic pathway leading to the destruction of damaged or redundant cellular components within vacuoles that allows for the maintenance of cellular homeostasis and the recycling of macromolecules into energy-rich metabolites (8). Autophagy has been shown to be crucial for bioenergetic metabolism during immune responses, notably in T cells (9, 10). Moreover, autophagy can occur in the form of xenophagy to eliminate invading intracellular pathogens (11, 12). Hence, autophagy can be expected to play a role in the immune response against SARS-CoV-2 infection and to reduce the severity of COVID-19 manifestations due to the elimination of SARS-CoV-2 in infected cells (13).

Autophagy is tightly regulated. Acyl CoA binding protein (ACBP), also called diazepam binding protein (DBI), is a phylogenetically conserved protein that binds to activated fatty acids (14). Through shuttling of lipids between cellular organelles, intracellular ACBP fosters oxidative phosphorylation and autophagy (15, 16). However, ACBP is also secreted and can be found in the circulation. When present in the extracellular milieu, ACBP inhibits autophagy and promotes appetite (17). High circulating ACBP levels have been observed in the context of aging and obesity (15, 17–19). Moreover, elevated plasma ACBP levels have been documented in patients at risk of developing cardiovascular diseases (20) and cancer (21).

ACBP has been studied in diabetes, obesity, and anorexia (15, 18, 20, 22). In plants, ACBP favors replication of some RNA viruses (23). Its role has been scarcely investigated in infectious diseases (24), including COVID-19. For this reason, we assessed circulating levels of ACBP and their association with disease severity in 1198 patients and controls from which plasma proteomics data were generated by the *Biobanque Québecoise de la COVID-19* (BQC-19) in Canada.

## Material and methods

### Participant and sample collection

BQC19 is a province-wide biobank established in Quebec, Canada, in March 2020 to foster collaborations on COVID-19 research by collecting, storing, and sharing of samples and data of people affected by COVID-19 (see bqc19.ca) (25). Participants were recruited upon contact with the healthcare system in the emergency room or during acute care hospitalization between March 2020 and August 2021. Participants were eligible to be enrolled to BQC19 if they had undergone PCR-testing for SARS-CoV-2. Participants with a positive PCR result were enrolled in the COVID+ group, and those with a negative test served as controls. Upon informed consent confirmation, medical charts containing comorbidity data were extracted, questionnaire were administered to participants and blood was collected at several timepoints. Clinical outcomes were assessed for all participants.

We analyzed proteomics and clinical data from 903 adults diagnosed with COVID-19 in the acute phase of infection, and 295 controls. All controls had negative COVID-19 test results. Controls were hospitalized for various reasons including COVID-19 negative respiratory infections, cardiovascular events, including embolic diagnoses, digestive symptoms or uncontrolled diabetes. For each patient, we considered the first available measurements of ACBP, i.e., the nearest in time to symptom onset. None among the participants was vaccinated against COVID-19 at the time of sample collection.

All participants gave written, oral, or substituted consent to participate in BQC-19. The Management framework of BQC-19 (including provision for oral or substituted consent) was approved by the center de recherche du centre hospitalier de l’université de Montréal (CR-CHUM) Research ethics board, and the present project was approved by the centre universitaire de santé McGill (CUSM) research ethics board.

The WHO Working Group on the Clinical Characterization and Management of COVID-19 criteria were used to categorize participants into mild, moderate or severe infections (26). Mild disease referred to participants requiring ambulatory care (emergency room visits without hospitalization), including people with asymptomatic presentation but detectable SARS-CoV-2 RT-PCR, or symptomatic but requiring little or no assistance. Moderate diseases encompass hospitalized patients who did not require oxygen therapy or received oxygen by mask or nasal cannula. Severe disease refers to hospitalized patients requiring oxygen delivered by positive airways pressure, intubation or mechanical ventilation, dialysis or extracorporeal membrane oxygenation. Participants who passed away during their hospitalization were included in a separate group when indicated.

### Blood sample processing and conservation

Whole blood was obtained through venipuncture using acid-citrate-dextrose vacutainer tubes, and plasma was separated by centrifugation at 750g, 10 min at room temperature. Isolated plasma was aliquoted and stored at −80°C until analysis.

### Plasma proteomics

Blood samples from a total of 1198 BQC19 participants were included for the plasma proteomics analysis. Proteomic profiles were assessed at SomaLogic using the SomaScan v4.0 proteomic platform. This platform provides measurements on 4701 unique human circulating proteins using 4987 Slow Off-Rate Modified Aptamers (SOMAmer reagents) and quantifies protein levels in the form of relative fluorescence units (RFUs) (27). Experimental process and data normalization including hybridization control normalization, intraplate median signal normalization, and plate scaling and calibration were performed as described (27–29).

In a subset of samples (n=33), plasma was obtained for ACBP levels quantification using a commercial kit following the supplier recommendations (Abnova, ELISA Kit cat. #KA6327, Taiwan).

### Circulating and cell-binding SARS-CoV-2 spike specific IgG quantification in plasma

SARS-CoV2 Spike-specific IgG levels were quantified by means of an ELISA and a cell-based ELISA (CBE) method as previously described (30, 31, 60). For the CBE, HOS cells were transfected to express SARS-CoV-2 spike proteins. Transfected cells were washed and incubated with diluted plasma (1:250). After washing, anti-human IgG, IgM and IgA antibody coupled to horseradish peroxidase (HRP) was added. After washing again, substrate was added, and light emission was quantified using a luminometer.

### Statistical analysis

GraphPad Prism 9.0 (GraphPad, CA, USA) was used to perform group comparisons and correlation analyses. Spearman’s rank correlation test was used to identify associations between 2 continuous variables. Mann-Whitney’s test and student t-test were used to compare levels of continuous variables between two independent groups, as appropriate. Kruskal-Wallis’ test, also known as one-way ANOVA test, was used to compare levels of continuous variables in more than 2 independent study groups. Distribution comparisons between COVID-19 patients and controls were performed using Chi-square tests. P-values <0.001 were considered significant for samples with n>250, and <0.05 for samples less than 250. A minimum value of 0.2 was considered for r values in significant associations assessed with the Spearman’s test. Logistic regression univariable models were used to generate Receiver operating characteristic (ROC) curves. SPSS (IBM, Chicago, IL, USA) was used for multivariable analyses.

### Ethics declaration

The BQC19 was approved by the research ethics boards (REB) of the Jewish General Hospital and the Centre Hospitalier de l’Université de Montréal (CHUM) in Montréal, QC, Canada. Informed consent was obtained from all participants. Data analyses for this project were approved by the research ethics board of the McGill University Health Centre (MUHC) from 2021 to 2024 (REB approval # 2021-7241).

## Results

### Participants characteristics and clinical outcome

A total of 1198 participants were included in the analysis. Among these participants, 903 were COVID-19 positive as determined by RT-qPCR tests (median age 59.5, range 18-99), encompassing 46% females and 54% males. In addition, 295 RT-qPCR COVID-19 negative hospitalized participants were included as controls (median age 49, range 20-89), encompassing 47% females and 53% males. Using WHO criteria (26), of the COVID-19 positive cohort (n=903), 259 were classified as mild, 384 as moderate, 236 as severe, and 24 as fatal. Slight differences in the frequency of participants with diabetes and cancer were observed between the COVID-19 and control groups. Details are included in **Table 1**.

**Table 1:** Participant characteristics.

COVID-19 Severity	COVID-19 positive (n=903)	COVID-19 negative Hospitalized controls (n=295)	P value
**Age** **Range**	59.5 (18-99)	49 (22-89)	0.03
**Sex: Women**	417 (46.2%)	142 (48.1%)	0.08
**Men**	486 (53.8%)	153 (51.9%)	
COVID-19 Severity					
Mild	259	N/A	
Moderate	384		
Severe	236		
Dead	24		
Obesity					
No	807 (89.37%)	267 (90.5%)	0.018
Yes	88 (9.75%)	23 (7.80%)	
Missing data	8 (0.89%)	5 (1.69%)	
Diabetes					
No	654 (72.43%)	227 (76.95%)	**<0.0001**
Yes	244 (27.02%)	63 (21.36%)	
Missing	5 (0.55%)	5 (1.69%)	
HIV					
No	887 (98.22%)	288 (97.63%)	>0.99
Yes	7 (0.78%)	2 (0.68%)	
Missing	9 (0.10%)	5 (1.69%)	
Cancer					
No	798 (88.37%)	244 (82.71%)	**<0.0001**
Yes (history or current)	103 (11.41%)	46 (15.59%)	
Missing	2 (0.22%)	5 (1.69%)	
Chronic obstructive pulmonary disease					
No	812 (89.92%)	268 (90.85%)	0.01
Yes	85 (9.41%)	22 (7.46%)	
Missing	6 (0.66%)	5 (1.69%)	
Cardiovascular disease					
No	419 (46.40%)	152 (51.53%)	0.002
Yes	484 (53.60%)	143 (48.47%)	
Missing	0 (0%)	0 (0%)	

N/A, not applicable. Statistically significant differences are indicated in bold.

### Plasma ACBP levels are elevated in COVID-19 patients and associate with severity

**Figure 1:**
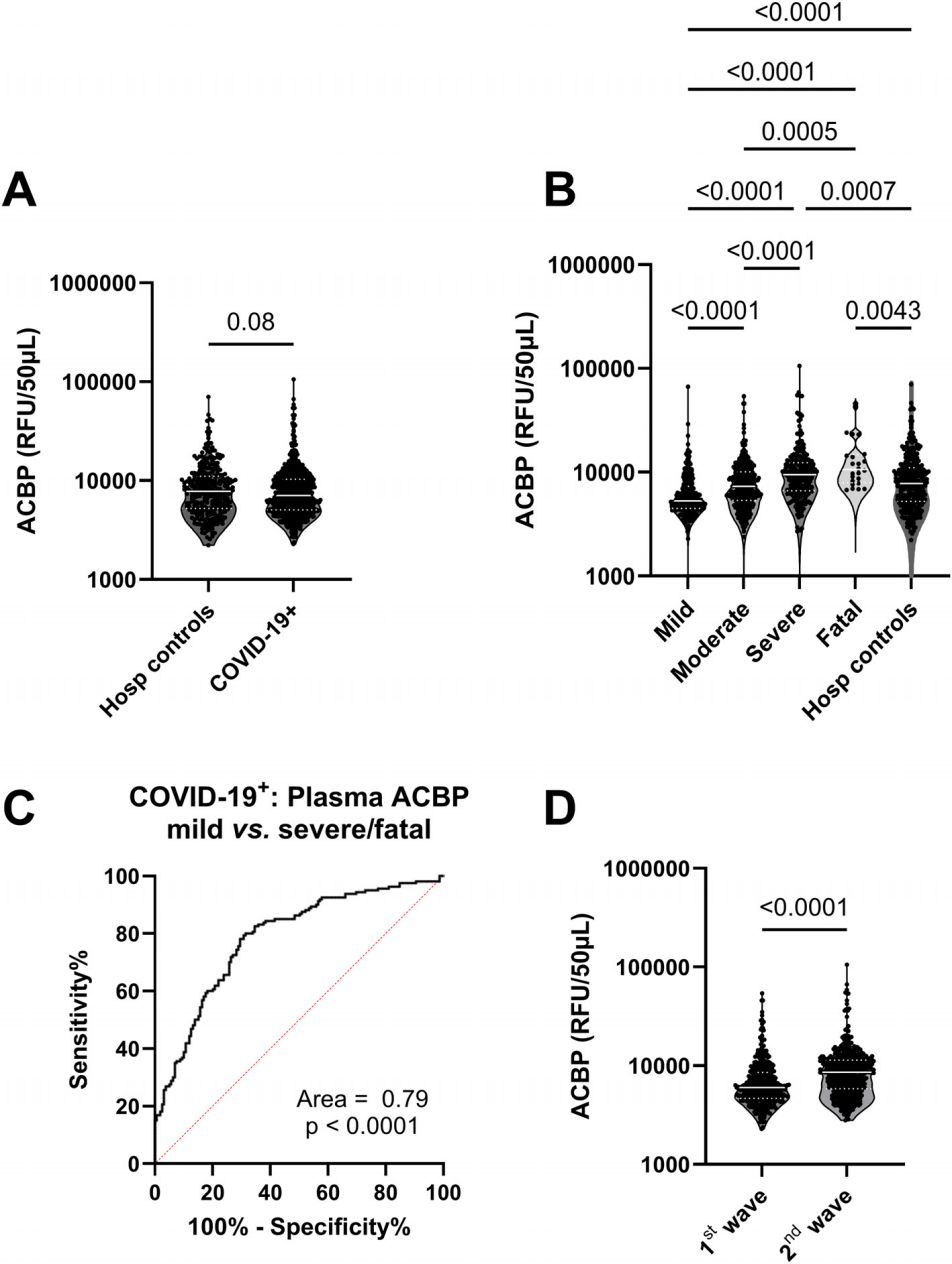
Plasma ACBP levels were elevated with severity in COVID-19 patients. **(A)** Comparison of plasma ACBP levels in hospitalized controls (n=295) and COVID-19 patients (n=903) (Mann Whitney’s test). **(B)** comparison of plasma ACBP levels according to disease severity in COVID-19 positive patients and hospitalized controls (mild n=267, moderate n=384, severe n=236, fatal n=24) (Kruskal Wallis’ test). **(C)** Receiver operating characteristic (ROC) curve comparing ACBP levels in mild vs severe and fatal COVID-19+ groups. **(D)** Comparison of plasma ACBP levels during the first wave (March 2020 – July 2020) with the second wave (August 2020 to August 2021, n=461) in the province of Quebec, Canada, (n=474) (Mann Whitney’s test). RFU, relative fluorescent units; ACBP, Acyl CoA Binding Protein.

Approximately 5000 plasma proteins were measured by SomaScan proteomics in COVID-19 infected and hospitalized control adult participants. Plasma ACBP levels were similar between COVID-19-positive participants and hospitalized controls (**Figure 1A**). However, in the COVID-19 positive group, plasma ACBP levels increased with disease severity, and the highest median ACBP levels were detected in the fatal group (p<0.0001) (**Figure 1B**). Severe COVID-19 groups also had significantly higher plasma ACBP levels compared to moderate and mild COVID-19 groups (p<0.0001 for both comparisons). Both severe and fatal groups had higher plasma ACBP levels than hospitalized controls (p<0.001 and 0.0043).

We were able to quantify plasma ACBP by ELISA and found a strong correlation between ACBP levels detected by SomaScan proteomics and ELISA (r=0.61, p<0.001) in 33 samples (**Supplementary Figure 1**). Also, we compared plasma ACBP levels assessed by ELISA in 33 COVID-19 patients, including 25 severe cases and 12 healthy controls (demographics detailed in **Supplementary Table 1**). We found higher levels of ACBP in severe COVID-19 than healthy controls (p=0.04) (**Supplementary Figure 1**).

Plasma ACBP concentrations were associated with age (r=0.29, p<0.001), with levels of GDF-15, a biomarker of aging (r=0.29, p<0.0001) (**Table 2**) (28), as well as with ferritin levels, which is an established marker of COVID-19 severity (r=0.3, p<0.001) (**Table 2**) (7). Multivariable analysis revealed that age, sex or the presence of comorbidities did not influence the association between ACBP levels and disease severity (**Table 3**). We evaluated the predictive ability of plasma ACBP to discriminate mild from severe COVID-19 states (including fatal cases) using receiver operating characteristic (ROC) curves (**Figure 1C**). The area under the curve (AUC) was estimated for ACBP levels and their predicted values by fitting regression models. Plasma ACBP concentration levels predicted COVID-19 disease severity with an AUC=0.79 ± 0.026 (p<0.0001).

**Table 2:** Plasma ACBP levels comparison with known markers of COVID-19 severity.

Plasma ACBP *vs.*	r	r 95% confidence interval	p value	n
**Age**	**0.29**	**0.23 to 0.35**	**<0.0001**	**903**
**Ferritin**	**0.3**	**0.23 to 0.36**	**<0.0001**	**903**
D-dimer	-0.016	-0.083 to 0.051	0.64	903
**GDF15**	**0.28**	**0.21 to 0.34**	**<0.0001**	**903**

Statistically significant correlations are indicated in bold.

**Table 3:** Multivariable comparisons.

		General Linear Model results		
		Wilks’ delta	F value	P value
Plasma ACBP levels and COVID-19 severity	Age	0.038	1.166	0.055
	Sex	0.947	4.046	0.019
	Type of comorbidity	0.945	4.228	0.016

Multivariate analysis of variance (General Linea Model) test performed using SPSS Statistics with data from 903 participants with severity data. P value < 0.001 was considered significant.

All other 5000 plasma proteins were compared between patients with mild and severe/fatal disease presentation using multiple Mann-Whitney’s tests. ACBP was found as one of the 712 proteins best associated with severity as shown by being one of the proteins with the most significant q and p values (-log_10_ 118.2; p<0.00001). Of note, the Q-values were less significant for IL-6 or CRP (-log_10_ 35.81 and 108.3, respectively, p<0.00001 for both), which are two common biomarkers of COVID-19 severity (4, 32) (**Supplementary Table 2**).

### Plasma ACBP levels were higher during the second wave of COVID-19

Two waves of COVID-19 cases were observed during the study period, the first between March 2020 and August 2020 and the second between September 2020 and May 2021 in Quebec, Canada (https://www.inspq.qc.ca/en/node/34836) (33). The first was dominated by B1.1 ancestral subtypes of SARS-CoV-2 while the second one was predominantly due to the α variant of SARS-CoV-2 (33). Interestingly, plasma ACBP levels were more elevated in the plasma of COVID-19 patients collected during the second wave as compared to the first one (8591 vs. 5999 RFU/50µL, p<0.0001) (**Figure 1D**).

COVID-19 patients were older during the first wave compared to the second one (66.9 vs. 57.4, p<0.001) (**Supplementary Figure 2**). This appears important because normal aging (in apparently healthy individuals) is associated with an increase in ACBP levels (15, 34). However, multivariable analyses showed that the variation in plasma ACBP concentrations was independent of age, comorbidities and sex in the two collection periods.

### Higher ACBP levels in severe COVID-19 patients independently of comorbidities

We then assessed whether comorbidities were associated with further elevation of ACBP levels in COVID-19 patients. The most common comorbidities observed in COVID-19 infected patients (n=903) were cardiovascular disease (CVD) (n=483, 53.4%), diabetes (244, 27.0%), cancer (16, 1.8%), obesity (89, 9.9%), chronic obstructive pulmonary disease (COPD) (84, 9.3%) and infection by human immunodeficiency virus (HIV) (7; 0.77%) (**Table 1**).

**Figure 2:**
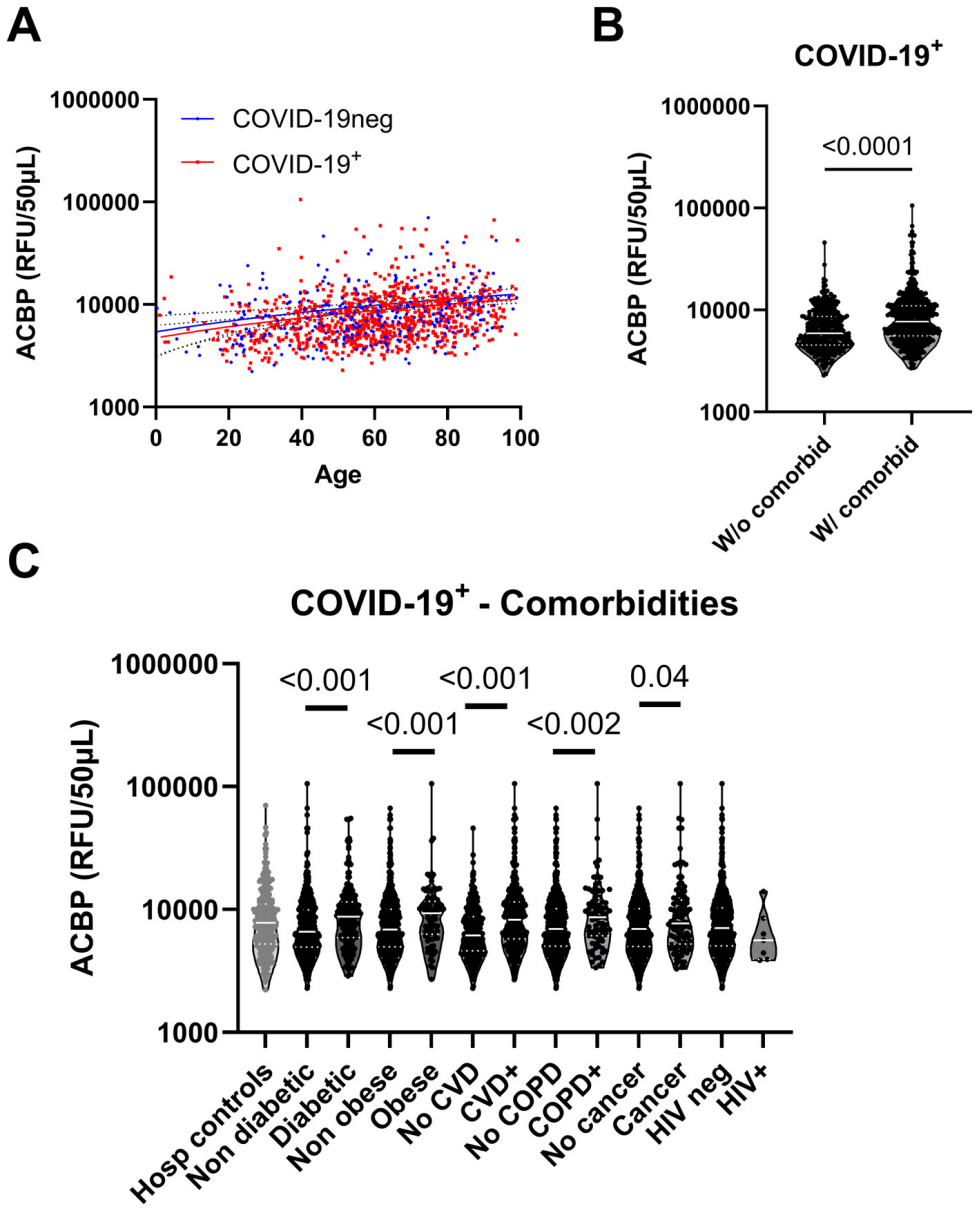
Plasma ACBP levels were elevated in COVID-19 independently of comorbidities. **(A)** Plasma ACBP levels association with age in the COVID-19 negative and COVID-19 positive groups (Spearman’s test). **(B)** Comparison of plasma ACBP levels between COVID-19^+^ groups without (n=348) or with any comorbidity (n=587) (Mann Whitney’s test). **(C)** Comparison of plasma ACBP levels in COVID-19 negative participants and COVID-19 groups based on their comorbidity (Mann Whitney’s test). CVD, cardiovascular disease; COPD, chronic obstructive pulmonary disease; HIV, human immunodeficiency virus.

Plasma ACBP concentrations correlated with age, a known co-factor of COVID-19 severity, in both COVID-19-positive patients and COVID-19-negative controls (r=0.29, p<0.001 and r=0.28, p<0.001 respectively) (**Figure 2A**). SARS-CoV-2-infected patients with comorbidities had higher plasma ACBP levels compared to those without known comorbidities (**Figure 2B**). Thus, COVID-19 patients with diabetes, obesity, CVD, or COPD had significantly higher plasma ACBP levels (p<0.001 for all comparisons) than patients without any of these comorbidities (**Figure 2C**). Although ACBP levels are elevated in people with HIV (PWH) (24), we observed similar ACBP levels in PWH with COVID-19 and HIV-uninfected COVID-19 patients. As a caveat, only 7 PWH participants were included in the present study.

In COVID-negative hospitalized controls, patients with cardiovascular diseases, diabetes or cancer had higher levels of ACBP compared to those without these condition (**Supplementary Figure 3**).

### ACBP levels correlate with inflammation and inversely correlate with cellular immunity

We then assessed whether inflammation would be associated with plasma ACBP levels. Plasma ACBP concentrations correlated with the elevated neutrophil/lymphocyte ratio (r=0.29, p<0.0001), which is an established marker of inflammation and severity (35, 36). In contrast, neither CD4 counts nor CD8 counts, which were measured in a subset of participants, were associated with circulating ACBP levels (**Table 4**).

**Table 4:** Plasma ACBP levels vs inflammation markers in COVID-19 participants.

Plasma ACBP *vs.*	r	r 95% confidence interval	P value	Number of XY Pairs
**Lympho count**	**-0.23**	**-0.30 to -0.15**	**<0.0001**	**695**
Mono count	0.004	-0.073 to 0.081	0.92	695
**Neutro count**	**0.21**	**0.14 to 0.28**	**<0.0001**	**695**
**Neutro/Lympho**	**0.29**	**0.22 to 0.36**	**<0.0001**	**694**
CD4 count	-0.017	-0.094 to 0.059	0.65	695
CD8 count	-0.018	-0.094 to 0.059	0.64	695
CRP	0.15	0.088 to 0.22	<0.0001	903
IL-1β	0.07	0.0042 to 0.14	0.032	903
**IL-6**	**0.42**	**0.36 to 0.47**	**<0.0001**	**903**
IL-8	0.099	0.034 to 0.16	0.002	903
**TNF-α**	**-0.32**	**-0.37 to -0.25**	**<0.0001**	**903**
**IL-21**	**-0.28**	**-0.34 to -0.22**	**<0.0001**	**903**
**IFN-γ**	**-0.23**	**-0.24 to -0.17**	**<0.0001**	**903**

Statistically significant correlations are indicated in bold.

Of note, circulating ACBP levels were strongly associated with markers of innate inflammation, such as C-reactive protein (CRP) and interleukin (IL)-6, but not with IL-1β or IL-8 (**Table 4**). However, plasma ACBP levels inversely correlated with several markers of lymphoid immune responses including TNF-α and IFN-γ. Moreover, ACBP anticorrelated with IL-21, which is participates to the crosstalk between CD4 and CD8 T cells and promotes antibody maturation and secretion (37) (**Table 4**).

In conclusion, ACBP levels correlate with surrogate markers of inflammation and anticorrelate with markers of T cell-mediated immune responses.

### Plasma ACBP levels correlate with circulating anti-SARS-CoV-2 antibody levels

To assess whether circulating ACBP levels were associated with anti-SARS-CoV-2 immune response, we compared ACBP levels with two measures of SARS-CoV-2 specific antibodies in plasma. Participants were enrolled early during their infection, at the time of initial healthcare contact. Despite this early time point, most participants (61.6%) already possessed circulating antibodies against the spike protein (see details in **Supplementary Figure 4**).

When all seropositive and seronegative participants were considered, plasma ACBP concentrations correlated positively with the titers of spike-specific IgA and IgM (**Table 5**; **Supplementary Figure 5**), as well as with circulating Spike-RBD antibody levels detected by several methods such as ELISA and antibody binding to Spike expressing cells.

**Table 5:** Association between plasma ACBP and anti-SARS-CoV-2 antibody levels in all participants.

Plasma ACBP *vs.*		r value	r 95% interval	p value	n
ELISA response against Spike RBD	IgG	0.082	0.016 to 0.15	0.012	903
	IgA	**0.21**	**0.15 to 0.27**	**<0.001**	**903**
	IgM	**0.2**	**0.13 to 0.26**	**<0.001**	**903**
	**Total Igs**	0.093	0.027 to 0.16	0.004	903
**Cell binding assay (full Spike protein)**	**IgG**	0.14	0.074 to 0.21	<0.001	886
	**IgA**	0.19	0.12 to 0.25	<0.001	883
	**IgM**	**0.22**	**0.16 to 0.29**	**<0.001**	**882**
	**Total Igs**	0.14	0.076 to 0.21	<0.001	885

Statistically significant correlations are indicated in bold.

Interestingly, among SARS-CoV-2-seropositive patients, only the circulating levels of anti-RBD total IgG were associated with plasma ACBP levels (**Table 6**). All correlations were statistically significant when they were computed for the relationship between plasma ACBP and IgG, IgA, IgM, as well as for total antibodies capable of binding to spike expressing cells.

**Table 6:** Association between plasma ACBP and anti-SARS-CoV-2 antibody levels in seropositive participants.

Plasma ACBP *vs.*		r value	r 95% interval	p value	n
ELISA response against Spike RBD	IgG	0.18	0.099 to 0.26	<0.001	572
	IgA	0.075	-0.028 to 0.18	0.141	390
	IgM	0.15	0.046 to 0.25	0.004	383
	**Total Igs**	**0.21**	**0.13 to 0.29**	**<0.001**	**595**
**Cell binding assay (full Spike protein)**	**IgG**	**0.29**	**0.20 to 0.36**	**<0.001**	**541**
	**IgA**	**0.2**	**0.088 to 0.31**	**<0.001**	**298**
	**IgM**	**0.27**	**0.16 to 0.37**	**<0.001**	**311**
	**Total Igs**	**0.28**	**0.20 to 0.36**	**<0.001**	**579**

Statistically significant correlations are indicated in bold.

### Correlations between ACBP levels and autophagy-relevant markers

Extracellular ACBP is a well-known inhibitor of autophagy (38, 39). Autophagy is a tightly regulated pathway involving multiple factors from the autophagy protein (ATG) family. We compared circulating levels of the autophagy inhibitor ACBP, with autophagy involved proteins detected by SomaScan proteomics such as the ATG proteins (ATG3, ATG4B, ATG5, ATG7) and other established effectors of autophagy such as Beclin-1 (BECN1), galectins 3 and 9 (40), as well as with the lipidated form of microtubule-associated proteins 1A/1B light chain 3B (LC3II) (**Table 7**). Plasma ACBP concentrations positively correlated with ATG 3, ATG5 and LC3II, but negatively correlated with ATG4B, BECN1 and galectin 8 in participants with COVID-19.

**Table 7:** Plasma ACBP vs. autophagy markers in COVID-19 participants.

Spearman r	r	95% confidence interval	P value	Number of XY Pairs
**ATG3**	**0.15**	**0.084 to 0.22**	**<0.0001**	**903**
**ATG4B**	**-0.22**	**-0.28 to -0.15**	**<0.0001**	**903**
**ATG5**	**0.35**	**0.29 to 0.40**	**<0.0001**	**903**
**ATG7**	**-0.37**	**-0.43 to -0.31**	**<0.0001**	**903**
**BECN1**	**-0.4**	**-0.46 to -0.34**	**<0.0001**	**903**
**LC3II**	**0.58**	**0.53 to 0.62**	**<0.0001**	**903**
Galectin 3	-0.021	-0.088 to 0.046	0.52	903
**Galectin 8**	**-0.27**	**-0.33 to -0.20**	**<0.0001**	**903**

Statistically significant differences are indicated in bold.

Altogether, these results suggest a link between the COVID-19 severity-related elevation of ACBP and autophagy in circulating leukocytes.

## Discussion

The overarching conclusion of this study is that plasma concentrations of the autophagy checkpoint ACBP are associated with COVID-19 severity, independently of the presence of comorbidities. We observed higher levels of ACBP in the plasma of patients collected during the second wave (August 2020 to August 2021) of the COVID-19 pandemic compared to the initial wave period (March 2020 to July 2020). This difference was independent of age, comorbidities and vaccination status (because none among the participants had received vaccination at the time of study). Moreover, both waves were primarily caused by genetically close ancestral SARS-CoV-2 strains. We previously reported that the second wave patients were younger (average 57.4 years of age) compared to those of the first wave (66.9 years of age) (28). However, ACBP levels were higher during the second wave, in those younger participants. and multivariable analysis showed an independence of age. Hence, age differences are unlikely to be responsible for the observed shifts in plasma ACBP levels. In this context, however, ACBP levels correlated with those of GDF-15, a biomarker of aging and mitochondrial dysfunction. Accordingly, we have previously demonstrated that GDF-15 is linked to COVID-19 severity as well (28). These results complement previous studies using proteomics to identify circulating biomarkers of COVID-19 severity (41, 42). Interestingly, in their supplementary data, Roh et al. showed that plasma ACBP were linked with cardiovascular complications in COVID-19 patients with different severity levels (43).

Extracellular ACBP has been implicated in the pathogenesis of several inflammatory conditions such as obesity (18), diabetes (19), cardiovascular disease (20) and aging (15). Interestingly, all these conditions are also risk factors of severe COVID-19, as this has been reported in several meta-analyses (3, 44–47). Our study revealed an independent association between COVID-19 severity and ACBP levels. Although ACBP blockade in mouse models prevented obesity (38), the causality of elevated circulating ACBP levels on COVID-19 severity will have to be confirmed in future studies.

In PWH, high ACBP levels are associated with inflammatory markers including the neutrophil/lymphocyte ratio, CRP, IL-1β, IL-6 and IL-8 (24). In contrast, in COVID-19 patients, ACBP plasma concentrations correlated with the neutrophil/lymphocyte ratio, CRP and IL-6 but not IL-1β and IL-8. Interestingly, in COVID-19 patients, markers of immune response such as TNF-α and IFN-γ were inversely associated with plasma ACBP levels. Moreover, an inverse correlation was also observed with IL-21 levels. Previous work has shown that autophagy contributes to energy metabolism in virus-specific cytotoxic T-cells (36, 37, 57, 58). In the context of chronic infections, the production of IL-21 stimulates lipophagy, which is a cargo-specific form of autophagy, in CD8 T-cells, hence stimulating their capacity to destroy virus-infected cells (1, 14, 15). Therefore, we speculate that extracellular ACBP might suppress autophagy in T cells, thereby compromising antiviral cellular immune responses.

We assessed the influence of plasma ACBP on SARS-CoV-2 specific immune response with anti-SARS-CoV-2 antibody levels. We observed a marked correlation between plasma ACBP levels and circulating antibodies recognizing the Spike protein from SARS-CoV-2. Antibody binding to infected cells has been shown to promote inflammation and disease progression, a phenomenon that is referred to a antibody dependent enhancement (ADE) (48), which might also foster ACBP release. However, ADE was not observed during COVID-19 (49). Hence, future mechanistic studies must determine possible causal relationship between plasma ACBP and antibody levels.

The relationship between ACBP and autophagy is complex and bidirectional. On one hand, intracellular ACBP can be actively secreted by cells through an autophagy-dependent, atypical mechanism (22, 38, 50). On the other hand, extracellular ACBP inhibits autophagy through an action on gamma-amino butyric acid (GABA) receptors containing the γ2 subunit (39, 51). We observed a strong positive correlation (r=0.58) between plasma ACBP levels and the abundance of LC3II in circulating leukocytes. LC3II is a dynamic autophagy marker that becomes overabundant when autophagy is stalled (52, 53). In contrast, we found a negative correlation (r=-0.40) between ACBP and leukocyte BECN1, which is the pathognomonic subunit of the autophagy-initiating phosphoinositide 3-kinase complex (54). This suggests an association between high extracellular ACBP concentrations and altered autophagic flux in the context of COVID-19. Insufficient autophagy might compromise the fitness of immune cells and the clearance of SARS-CoV-2 (55, 59). However, this conjecture requires further investigation in suitable animal models.

Although our study involved a total of 1198 COVID-19 patients and control participants, this work has several limitations. We only correlated COVID-19 severity with ACBP plasma concentrations in the first available plasma sample. It will be important to investigate dynamic changes in ACBP levels during viral infection and disease evolution to gain more insights into the possible pathogenic role of ACBP.

## Conclusions

High plasma ACBP levels were associated with COVID-19 severity independent of age, sex and comorbidity. The associations between circulating ACBP levels, inflammation and anti-SARS-CoV-2 antibody responses warrant further investigation on the role of extracellular ACBP during viral infection. Extracellular ACBP might play a direct role on SARS-CoV-2 pathogenesis through inhibition of immune responses. Hence, our results suggest that targeting extracellular ACBP (51) and other autophagy-enhancing strategies could reduce disease progression and favor SARS-CoV-2 immune control. Future studies should assess the influence of ACBP on disease severity in vaccinated individuals and explore the possible role of ACBP in other acute infections such as influenza (56).

## Data Availability

The original contributions presented in the study are included in the article/**Supplementary Material**, further inquiries can be directed to the corresponding author or the BQC-19.
