# Supplementary material for: Extracellular acyl-CoA-binding protein as an independent biomarker of COVID-19 disease severity
Source: Front Immunol. 2025 Jan 6;15:1505752. doi: 10.3389/fimmu.2024.1505752 (PMC11743960; doi:10.3389/fimmu.2024.1505752)
Supplement: Supplementary file 1 [file DataSheet1.docx]

Isnard et al.

**Supplementary data**

**Extracellular acyl-CoA-binding protein (ACBP) as an independent biomarker of COVID-19 disease severity**

**Supplementary table 1: demographics of participants for ELISA quantification of plasma ACBP levels**

| **COVID-19 Severity** | **COVID-19 positive**  **(n=33)** | **Healthy controls (n=12)** |  |
| --- | --- | --- | --- |
| **Age**  **Range** | | 61  (49-75) | 66  (28-89) |
| **Sex: Women**  **Men** | 9 (27.3%)  24 (72.7%) | 3 (25%)  9 (75%) |  |
| **COVID-19 Severity** |  |  |  |
| Mild | 2 (6%) |  |  |
| Moderate | 6 (18.2%) |  |  |
| Severe | 25 (81.8%) |  |  |
| Dead | 0 |  |  |

**Supplementary table 2, attached: comparison of Q value for all SomaScan identified protein between Mild and Severe/Fatal groups.**

Multiple Mann-Whitney’s tests were performed between mild and Severe/Fatal groups. P value and Q values are indicated for each identified proteins.

**Supplementary figure 1: Validation of SomaScan results by ELISA**


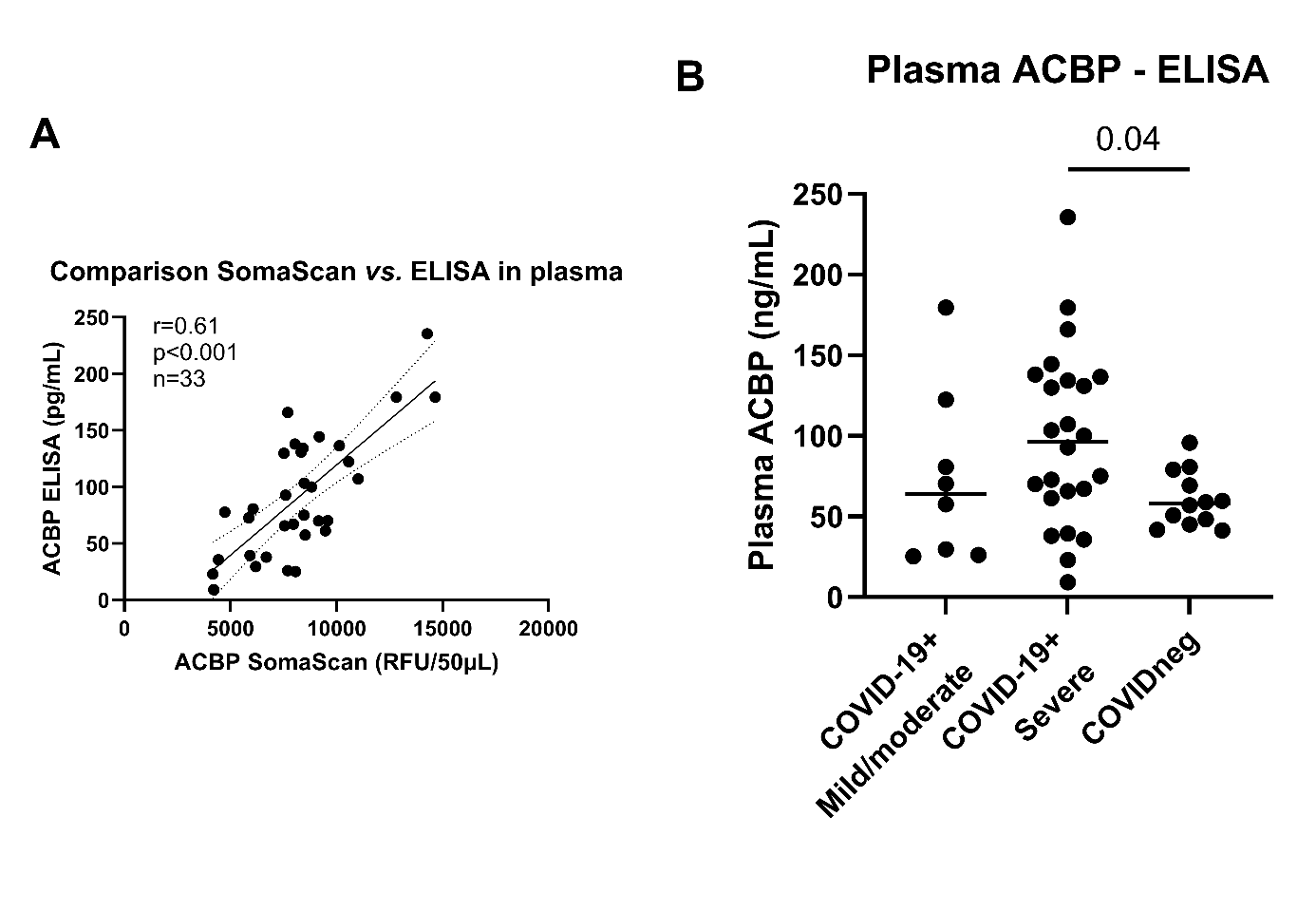


A: Plasma levels of ACBP quantified by SomaScan and ELISA were compared in 33 samples. Spearman’s correlation test. B: Comparison of plasma ACBP levels assessed by ELISA in COVID-19^+^ severity groups and 12 COVID-19negative healthy participants. Kruskal-Wallis’s test.

**Supplementary figure 2: age distribution during the two COVID-19 waves in Quebec**

**Supplementary figure 3: Plasma ACBP levels assessed by SomaScan in COVID-19 negative hospitalized patients depending on their comorbidities.**

Mann-Whitney’s test. White bar shows median.

**Supplementary figure 4: SARS-COV-2 specific antibody levels in COVID-19 groups**


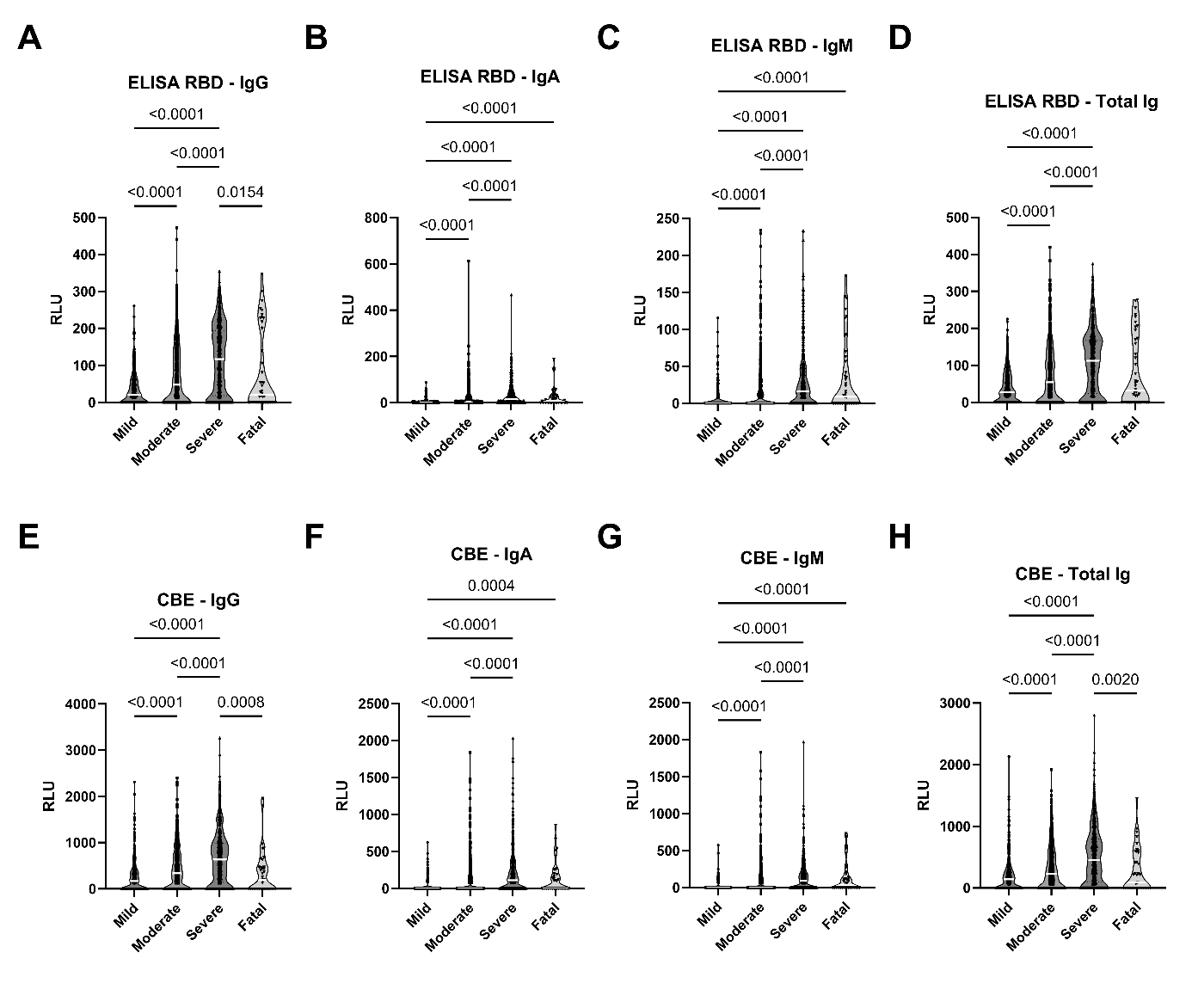


A-D: Anti-Spike-receptor binding domain (RBD) levels of IgG (A), IgA (B), IgM (C) or total immunoglobulins (D) quantified by ELISA in COVID-19 severity groups. E-H: Anti-spike levels of IgG (E), IgA (F), IgM (G) or total Ig (H) quantified by cell-based ELISA in COVID-19 severity groups. RLU = relative luminescence units. Mann-Whitney’s test. White bar shows median.

**Supplementary figure 5: Correlations plots of plasma ACBP levels and anti-SARS-CoV-2 antibodies in all participants.**


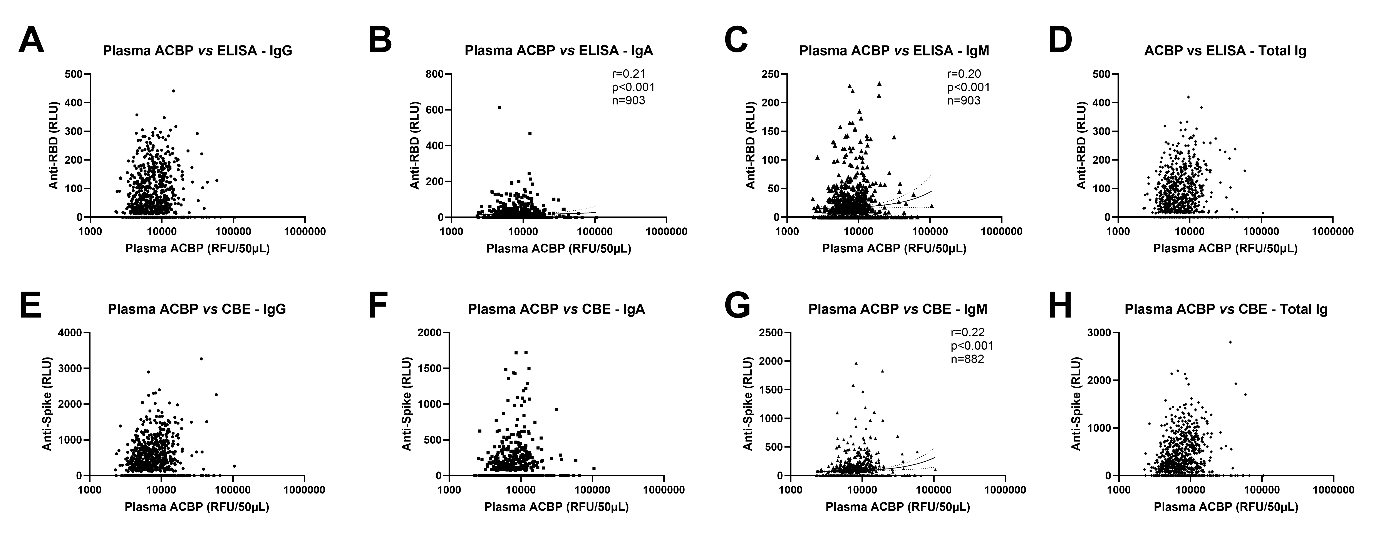


A-D: Correlation plots of plasma ACBP quantified by SomaScan with anti-Spike-receptor binding domain (RBD) levels of IgG (A), IgA (B), IgM (C) or total immunoglobulins (D) quantified by ELISA in COVID-19 severity groups. E-H: Correlation plots of plasma ACBP quantified by SomaScan with anti-spike levels of IgG (E), IgA (F), IgM (G) or total Ig (H) quantified by cell-based ELISA in COVID-19 severity groups. RLU = relative luminescence units, RFU = relative fluorescence units. Spearman’s test. Linear regression shows slope and 95% confidence internal in dotted lines.
